# The effectiveness of low trans-fatty acids dietary pattern in pregnancy and the risk of gestational diabetes mellitus

**DOI:** 10.22088/cjim.10.2.197

**Published:** 2019

**Authors:** Seideh-Hanieh Alamolhoda, Masoumeh Simbar, Parvin Mirmiran, Parvaneh Mirabi

**Affiliations:** 1 Midwifery and Reproductive Health Research Center, Department of Midwifery and Reproductive Health, school of Nursing and Midwifery, Shahid Beheshti University of Medical Sciences, Tehran, Iran; 2 Endocrine Research Center, Research Institute for Endocrine Sciences, Shahid Beheshti University of Medical Sciences, Tehran, Iran; 3 Infertility and Reproductive Health Research Center, Health Research Institute, Babol University of Medical Sciences, Babol, Iran

**Keywords:** Gestational diabetes mellitus, Trans-fatty acids, pregnancy

## Abstract

**Background:**

Gestational diabetes mellitus (GDM) is a common disorder in pregnancy. The association of trans fatty acids (TFA) intake and risk of GDM have been reported; It remains unclear whether dietary TFA can influence GDM risk. We examined the effect of low TFA dietary intakes during pregnancy on risk of GDM.

**Methods:**

This randomized controlled trial was performed on 800 pregnant women who were randomly divided into 393 intervention and 407 comparison groups with gestational age ≥7 weeks. In the intervention group, the diet of pregnant women was designed in such a way that their daily intake of TFA content was less than1% but in control group, the daily intake of TFA content was not changed. The dietary intake was assessed using a 24-hour dietary recall questionnaire for three non-consecutive days at the beginning of the pregnancy before week 7, and at 13, 25 and 35 weeks. Diagnosis of GDM was performed using a 3-hour glucose tolerance test with 100 g glucose at 24-28 weeks of gestation.

**Results:**

14 women in the intervention group (5%) and 31 women in the control group (8%) were diagnosed with GDM. Chi-square test did not show any significant difference between two groups (P=0.08). Cox model was used and the variables were examined in four multivariate models that none of the modals showed a statistically significant difference between the two groups regarding the incidence of GDM.

**Conclusion:**

It seems that the diet with low trans-fatty acid content has no effect on the incidence of GDM.

Gestational diabetes is one of the adverse effects of pregnancy that is diagnosed with increased blood sugar in pregnant women, for the first time, during pregnancy (1, 2). Many risk factors are involved in this disease incidence, and in recent decades, many attempts have been made to identify further risk factors to prevent gestational diabetes (3, 4). One risk factor that has recently been taken into consideration is mothers’ diet during pregnancy (5). A component of diet that can affect glucose homeostasis and insulin action in mothers is the trans-fatty acids in the mother's diet (6). In a study conducted by Radesky et al. in 2008, it was stated that the consumption of trans-fatty acids increased the risk of developing gestational diabetes (7). 

In 2011, Ley et al. showed that reduced consumption of unsaturated fatty acids and increased intake of trans-fatty acids intensifies the risk of developing gestational diabetes (8). However, Bowers et al., stated that the use of trans-fatty acids does not affect insulin and glucose homeostasis (9).

Despite the inconsistencies in the results of studies, so far no study has been done in Iran to evaluate the effect of trans-fatty acids in pregnancy and its effect on gestational diabetes mellitus. Hence the researcher attempted to conduct a study on the effect of diet with low intake of trans-fatty acid content on gestational diabetes mellitus in pregnant women who referred to Shahid Beheshti University of Medical Sciences in 2014-2017.

## Methods

**Study Population: **This study is a randomized controlled trial that was conducted in Tehran from December 2014 to February 2017 after approval by ethical committee of Shahid Beheshti University of Medical Sciences under the following code of practice IR.SBMU.ries.Rec.1394.92 (IRCT2016092729902N3). In this study, 1016 pregnant women that fulfilled the inclusion criteria were registered after obtaining written informed consent; they referred to Shahid Beheshti University of Medical Sciences for receiving prenatal care. The criteria for entering the study were: gestational age ≥7 weeks; singleton pregnancy; 24>BMI>18, age range of 18-35; the number of pregnancy was less than 3; abortion less than 2; no history of gestational diabetes in previous pregnancies; no history of infant Macrosomia; no history of fetal death; no history of diabetes in first-degree relatives; no chronic diseases such as diabetes and hypertension; no consumption of cigarette, alcohol or drugs. During the study, 133 pregnant women were excluded from the study because they did not attend the health centers, 49 pregnant women were excluded because of their reluctance to continue with the study and 54 pregnant women were excluded since they did not follow the diet designed for them properly. Ultimately, 800 pregnant women were studied in the present study.

In the first appointment with pregnant women, the purpose of the study and the method of work were fully explained. Then, demographic and midwifery questionnaires were filled in for each pregnant woman through an interview. The pregnant women were divided into two groups of control and intervention by random number tables.

**Data Collection and Evaluation:** The pregnant women information was collected through face-to-face interview using organized questionnaires. The questionnaires consisted of three parts. 1. A demographic questionnaire examining the age, occupational status and education of pregnant women. Information obtained from this questionnaire was important because studies have shown that age, employment and education status of pregnant women can affect pregnancy outcomes such as diabetes (2, 10). 2. Midwifery questionnaire that provided information about the gestational age at the first appointment, the number of pregnancies, the number of abortions, the gestational age at the next referrals, the pre-pregnancy BMI, fasting blood glucose, fasting blood glucose at gestational age of 24-28 weeks; and one, two, and three hours after receiving 100 grams of glucose syrup. 3. A 24-hour dietary recall questionnaire assessed dietary information of pregnant women. The questionnaire includes breakfast, snack, lunch, snack, dinner, and snack before bedtime, and examines the details of pregnant women’s diet during the last 24 hours. The questionnaire was filled in through interviews in four stages at the beginning of pregnancy (before intervention and less than 7 weeks), 13 weeks, 25 weeks and 35 weeks of gestation in three non-consecutive days (one non-working day and two working days). The first dietary recall questionnaire was filled through interviews at the health center, and it usually lasted from 20 to 30 minutes, and subsequent questionnaires was filled through phone conversation. Each interview lasted about 20 minutes. The pregnant women were asked about the daily food items in detail, and then the details were transferred to gram and entered the questionnaire. The interviews were conducted by five nutritionists; and a midwife supervised the performance of the group. Prior to study conduction, all interviewers received training for three days on how to conduct interviews and complete the 24-hour dietary questionnaire, how to collect nutritional information and how to complete the other questionnaires.

**Nutrition Intervention: **For all pregnant women who participated in the study, a unique daily diet was designed based on their age, height, pre-pregnancy weight and daily physical activity.

To reduce trans-fatty acids to less than 1% of total daily energy intake of pregnant women in the intervention group, a food basket was provided for pregnant women including olive oil for cooking, 0% dairy products and nuts for the necessary fat content of the diet. The consumption of any kind of fast foods, deep-fried foods and processed protein products such as sausages and hotdogs was also prohibited for pregnant women. For pregnant women, the control group also was provided with a food basket containing liquid oil, 1.5% dairy products, and pregnant women in this group were recommended to refrain from fast foods and deep-fried foods and processed protein products. All pregnant women were also given an alternative list of foods in addition to the diet so that they could replace any item in the designed diet list that they were reluctant to use with a similar item on the alternative list. The dietary intake of pregnant women was evaluated using the US Department of Agriculture and the Iranian food composition table (11, 12). All dietary recall questionnaires were checked by the researcher and any ambiguity was examined and resolved via a proposal of question from pregnant women.

**Midwifery Information: **The pregnant women in both groups received routine nursing care. The gestational age of pregnant women was measured from the first day of their last menstrual cycle and via medical ultrasound at gestation of less than 12 weeks. The pregnant women up to 28 weeks received routine pregnancy care monthly, from 28 to 36 weeks, twice a month, and then up to the end of pregnancy they received weekly care.

**Data collection and outcome measures:** The primary endpoint measure in this trial is glucose dysfunction, which will be assessed in terms of mean reduction in fasting plasma glucose levels from the time of the baseline assessment to the follow-up assessment (24-28 weeks) on a 100 gram oral glucose tolerance test.

**Fig 1:**
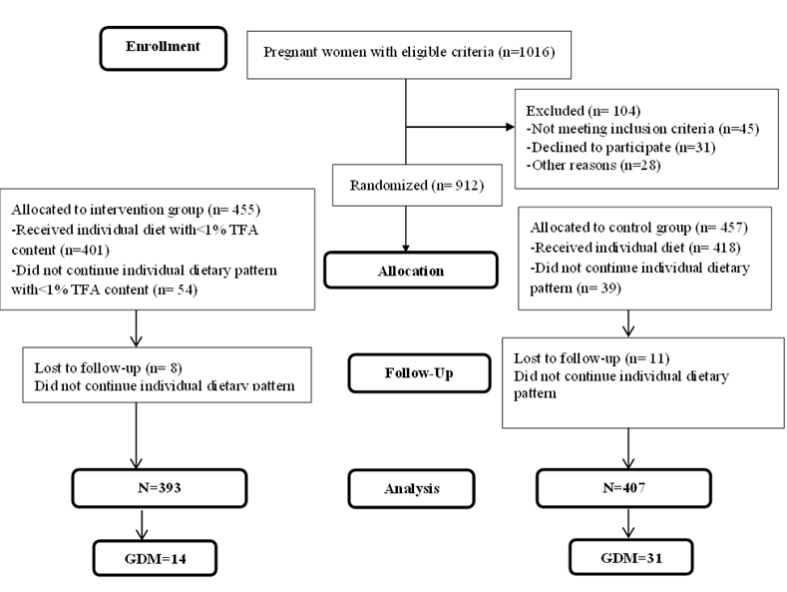
CONSORT follow chart

Blood glucose of all pregnant women was routinely examined at the beginning of pregnancy (in the first visit) and then at 24-28 weeks of gestation. Also, all pregnant women performed a-three hour glucose tolerance test at 100 g oral glucose at 28-24 weeks, then their blood glucose was examined one, two and three hours after receiving glucose syrup, and in the case that fasting blood glucose was more than 92, and one hour later, blood glucose was more than 180 given, and two hours later, it was more than 155, and three hours later, it was more than 140mg/dl, there women were diagnosed with gestational diabetes. Also, if two of these four blood tests were abnormal, the pregnant women were diagnosed with gestational diabetes mellitus (figure 1).

**Statistical Analysis: **Normality of all variables was investigated using Kolmogorov-Smirnov test and histogram chart. All of the variables studied were normal. Mean, standard deviation and frequency were calculated for all variables in both groups. In this study, to test the significance level between the two groups, t-test was used for quantitative variables and chi-square test was used for qualitative variables. 

The repeated measures test was used to evaluate the midwifery and nutritional changes during follow up. A logistic regression test was used to evaluate the effect of diet with trans-fatty acid content less than 1% on gestational diabetes mellitus. 

The Cox model was used to evaluate the effect of diet with trans-fatty acid content less than 1% over time. 

In this model, the study groups were considered as independent variables and gestational diabetes mellitus as dependent variable. 

Incidence rate and risk ratio (CI=95%) for gestational diabetes were calculated in both groups. 

The multivariate models for the two groups were based on age (years) and pre-pregnancy BMI (kg/m^2^) in the first model; age, pre-pregnancy BMI and the number of pregnancies in the second model; age, pre-pregnancy BMI, the number of pregnancies and fasting blood glucose at the beginning of gestation in the third model; age, pre-pregnancy BMI, the number of pregnancies, fasting blood glucose at beginning of gestation and fasting blood glucose at 24-28 weeks of gestation in the fourth model. The significance level was considered P<0.05.

## Results

Among the 1016 pregnant women who participated in this study, 113 pregnant women were excluded from the study because they refused to attend health centers, 49 pregnant women were excluded because their husbands were reluctant about their participation in the study and 54 pregnant women were excluded since they did not follow the diet designed for them properly. Finally, 800 pregnant women in the study were divided into comparison group with n=407 pregnant women and intervention group with n=393 women. The mean ages of pregnant women were 2.8±24.5 and the mean of pre-pregnancy BMI in pregnant women were 1.6±21.9kg/m^2^ and there was no significant difference between the two groups in terms of these two variables (P=0.31) and (P=0.34). 67.5% of participants had diploma and 67.3% of the pregnant women were housewives and there was no significant difference between the two groups in terms of these two variables (P=0.82) (table 1).

Statistical analysis showed that there was no significant difference between the two groups in terms of daily intake of energy, protein, carbohydrate and fat at the end of each quarter of pregnancy, and the two groups were completely homogeneous in terms of daily intake of macronutrients (P>0.05). There was a significant difference between the intervention and comparison groups regarding the daily trans-fatty acid intake (P<0.05) (table 2). In the intervention group, all pregnant women received less than 1% of the total energy intake, trans-fatty acids, while the average daily intake of trans-fatty acids by pregnant women in the control group was 7% of the total daily energy intake.

**Table1:** Baseline characteristics of the all participants via the intervention and control groups

**P-value** *	**Intervention** **(n=393)**	**Control** **(n=407)**	**All** **(n=800)**	
0.31	24.4(2.9)	24.6(2.7)	24.5(2.8)	Age(years)
0.34	22(1.72)	21.9(1.63)	21.9(1.61)	Pre pregnancy BMI(Kg/m^2)^
0.93	109.7(7.67)	109(7.36)	109.7(7.33)	blood pressure<7 weeks
0.82	272(68)	266(66)	538 (67)	House wives occupation n (%)
0.11	268(67)	272(68)	540(67)	High school education n (%)
0.12	299(76)	302(74)	601(75)	Nulli porous (%)
0.56	330(83)	347(83)	667(83)	Para 0 n (%)
0.23	362(89)	352(89)	714(89.3)	Abortion 0 n (%)

Data are presented as mean (standard deviation) for continuous variables and percentage for categorically distributed variables.

**Table2:** Dietary intake of participants of the intervention and control groups

		**Control** **(n=407)**	**Intervention** **(n=393)**	***P*** **-value**
Energy (kcal)	<7weeks	2083±241	2024±207	0.06
	13weeks	2024±61	2015±54	0.08
	25weeks	2352±53	2346±53	0.06
	35weeks	2457±50	2447±52	0.07
Carbohydrate (%)	<7weeks	50±3.81	51±3.21	0.06
	13weeks	53±3.32	55±2.55	0.07
	25weeks	52±3.13	53±2.52	0.06
	35weeks	53±3	54±2.62	0.07
Protein (%)	<7weeks	13±1.41	12±1.36	0.06
	13weeks	18±1.24	19±1.25	0.08
	25weeks	17±1.23	18±1.26	0.07
	35weeks	17±1.11	18±1.02	0.07
Total fat (%)	<7weeks	37±10	37±8	0.06
	13weeks	30±6.65	30±6.23	0.08
	25weeks	31±3.12	30±3.24	0.08
	35weeks	30±2.61	29±2.94	0.07
TFAs (%)	<7weeks	10±2.32	9±1.34	0.07
	13weeks	8±1.33	1±0.82	0.04
	25weeks	7±1.15	0.91±0.65	0.03
	35weeks	7±1.45	0.89±0.60	0.03

Data are presented as mean (standard deviation)

**Fig 2:**
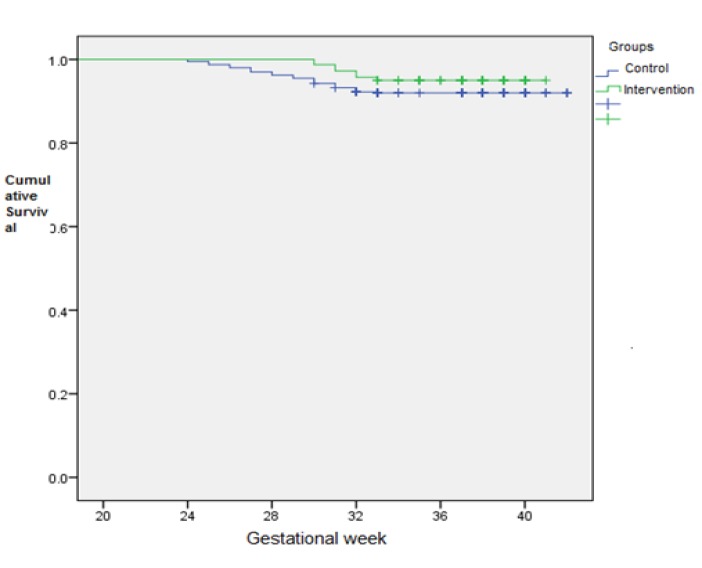
Kaplan-Meier plots for GDM in two intervention and control groups

Of the 800 pregnant women who participated in the study, 45 pregnant women were diagnosed with gestational diabetes of which 31 (8%) of them were in the comparison group and 14 (5%) others were in the intervention group. Chi-square test did not show a significant difference between the two groups (P=0.08). With regard to the difference in the incidence rate of gestational diabetes in both intervention and control groups, and also to investigate the effect of time factor in incidence of this adverse effect, Cox model was used and the variables were examined in four multivariate models that none of the modals showed a statistically significant difference between the two groups regarding the incidence of gestational diabetes (table 3). The Kaplan Meier chart showed that in comparison group, the onset of gestational diabetes mellitus in pregnant women occurs at gestation of 24 weeks, and in intervention group, it occurs at gestation of 30 weeks. In other words, a diet with trans-fatty acid content of less than 1% could have delayed the incidence of gestational diabetes in the intervention group of 6 weeks gestation. 95% of women with gestational diabetes mellitus were at gestational age of over 32 weeks in the intervention group; and in control group, only 8% of pregnant women with gestational diabetes were at gestational age of over 32 weeks, and 92% of them had developed gestational diabetes between 24-32 weeks of gestation (figure 2); diet with trans-fatty acid content of less than 1% increased the gestational age for the incidence of gestational diabetes mellitus in the intervention group.

**Table 3:** Hazard ratio (95% CI) for developing GDM based on dietary pattern ≤1% TFAs intake

**Models**	**Hazard ratios**	**CI (95%)**	**P-value**
Model.1 Model.2 Model.3 Model.4	0.60 0.58 0.68 0.92	0.34-1.05 0.34-1.01 0.39- 1.20 0.51- 1.60	0.07 0.06 0.07 0.08

Model.1: group was adjusted.

Model.2: group, age, pre pregnancy BMI and gravidity were adjusted.

Model.3: group, age, pre pregnancy BMI, gravidity and first trimester fasting blood sugar were adjusted.

Model.4: group, age, pre pregnancy BMI, gravidity, trimester fasting blood sugar and fasting blood sugar at 24-28 week were adjusted.

## Discussion

In this randomized controlled trial, the effect of diet with trans-fatty acid content of less than 1%, on the risk of gestational diabetes mellitus was investigated. The results of this study showed that although the incidence rate of gestational diabetes in the intervention group was 3% lower than in control group, the decrease was not statistically significant, moreover, Cox regression model did not show any significant difference in risk ratio between intervention and control groups (P>0.05). 

The results of this study were consistent with the results of the study by Lauszus et al., 2001 (13). In clinical trial study conducted by Lauszus et al. in Denmark, there was no significant difference in the risk ratio of gestational diabetes in pregnant women receiving a diet rich in fatty acids with pregnant women receiving a carbohydrate-rich diet. Lauszus et al. have stated that trans-fatty acids did not affect the probability of developing gestational diabetes and also had no effect on the incidence time of gestational diabetes. Lauszus et al stated that excessive intake of trans-fatty acids may be effective on insulin resistance, but this effect cannot lead to gestational diabetes.

In a clinical trial conducted by Louie et al. on 92 pregnant women in 2011, the risk of developing gestational diabetes in pregnant women receiving fiber-rich diets was reduced by 16% in proportion to the pregnant women receiving a diet rich in fatty acids and the protein (14). In the study of Louie et al., The mean age of gestational diabetes incidence in pregnant women that received diet rich in fiber was at 33 weeks and in pregnant women that received diet rich in fat and protein was at 28 weeks. Louie et al. stated that fiber in the diet could improve pancreatic function, reduce insulin resistance, and improve glucose homeostasis due to high levels of antioxidants. The results of the study by Louie et al. are inconsistent with the results of the present study. This contradiction can be due to several reasons. First, Louie et al. did not place any limitation on pre-pregnancy BMI of pregnant women who participated in the study and included pregnant women with a pre-pregnancy BMI of over 25kg/m^2^ as well. Overweight and obesity (BMI more than 25kg/m^2^) are the known risk factors in gestational diabetes mellitus (15). 

But in the present study, only pregnant women with a pre-pregnancy BMI of 19-25 kg/m^2^ were included in the study. Second, Louie et al. also included pregnant women with a history of gestational diabetes. The history of gestational diabetes is a known risk factor in the incidence of gestational diabetes (16). In the current study, the pregnant women participated in the study did not mention any history of gestational diabetes. Third, in the study by Louie et al., Cox's model was used to examine the effect of diet types on the incidence of gestational diabetes over time and the duration of the intervention was six months (the first and second trimester). In the present study, a regression model was used to evaluate the effect of trans-fatty acids on gestational diabetes mellitus and the duration of intervention was at least eight months.

Studies have revealed contradictory results regarding the effects of trans-fatty acids on insulin resistance. The difference in the results may be due to the difference in the population studied or the difference in the time duration of receiving trans-fatty acids or the difference in the type of intervention and method of work. Some studies indicated the effect of trans-fatty acids on insulin resistance, some other studies pointed to the inability of these fats to affect insulin resistance (14, 17-18). 

Other studies have also suggested that trans-fatty acids may interfere with glucose metabolism and result in glucose tolerance disorder and insulin resistance; this condition in pregnancy is itself a diabetic condition that can increase the risk of gestational diabetes incidence (19-22). Therefore, further studies are needed to examine the effect of trans-fatty acids on insulin resistance and on the incidence of gestational diabetes (17, 20).

One of the strong points about the present study is the design of a unique diet for each pregnant woman, and the diet for each group was only different in pregnant women’s daily intake content of trans-fatty acids. Moreover, in the present study, a nutritional intervention and the diet were provided for the pregnant women at gestational age of 7 weeks, and all pregnant women, along with the diet, were given an alternative list so that they could replace any item in the designed diet list that they were reluctant to use with a similar item on the alternative list that had the same nutritional value. 

Another strong point about this study was the supervision of the exact and accurate implementation of the diet designed for pregnant women at the end of each trimester via a 24-hour dietary recall questionnaire. Furthermore, in the present study, we considered all the risk factors of gestational diabetes as exclusion criteria of the research units so that we can examine the effect of a diet with a trans-fatty acid content of less than 1% on the incidence of gestational diabetes. The sample size of the present study is sufficient and suitable enough to accurately and exactly evaluate the effect of trans-fatty acids on the risk of gestational diabetes mellitus. So far, no clinical trials have been conducted in Iran to evaluate the effect of daily intake of trans-fatty acids on the incidence of gestational diabetes. 

One of the limitations of this study is the lack of blood samples from pregnant women in order to assess the serum level of trans-fatty acids, however, in the present study, 24-hour dietary recall questionnaire was used to evaluate trans-fatty acid intake that it is adequate enough for the exact measurement of trans-fatty acid intake (23).

Finally, this study showed that a diet with trans-fatty acid content of less than 1% could delay the incidence of gestational diabetes for 6 weeks that the same factor could reduce the risk of adverse effects such as polyhydramnios, macrosomia and consequently it reduces cesarean delivery.
